# Predicting Binding within Disordered Protein Regions to Structurally Characterised Peptide-Binding Domains

**DOI:** 10.1371/journal.pone.0072838

**Published:** 2013-09-03

**Authors:** Waqasuddin Khan, Fergal Duffy, Gianluca Pollastri, Denis C. Shields, Catherine Mooney

**Affiliations:** 1 Complex and Adaptive Systems Laboratory, University College Dublin, Dublin, Ireland; 2 Hussain Ebrahim Jamal Research Institute of Chemistry, International Center for Chemical and Biological Sciences, University of Karachi, Karachi, Pakistan; 3 Conway Institute of Biomolecular and Biomedical Science, University College Dublin, Dublin, Ireland; 4 School of Medicine and Medical Science, University College Dublin, Dublin, Ireland; 5 School of Computer Science and Informatics, University College Dublin, Dublin, Ireland; University of Alberta, Canada

## Abstract

Disordered regions of proteins often bind to structured domains, mediating interactions within and between proteins. However, it is difficult to identify a priori the short disordered regions involved in binding. We set out to determine if docking such peptide regions to peptide binding domains would assist in these predictions.We assembled a redundancy reduced dataset of SLiM (Short Linear Motif) containing proteins from the ELM database. We selected 84 sequences which had an associated PDB structures showing the SLiM bound to a protein receptor, where the SLiM was found within a 50 residue region of the protein sequence which was predicted to be disordered. First, we investigated the Vina docking scores of overlapping tripeptides from the 50 residue SLiM containing disordered regions of the protein sequence to the corresponding PDB domain. We found only weak discrimination of docking scores between peptides involved in binding and adjacent non-binding peptides in this context (AUC 0.58).Next, we trained a bidirectional recurrent neural network (BRNN) using as input the protein sequence, predicted secondary structure, Vina docking score and predicted disorder score. The results were very promising (AUC 0.72) showing that multiple sources of information can be combined to produce results which are clearly superior to any single source.We conclude that the Vina docking score alone has only modest power to define the location of a peptide within a larger protein region known to contain it. However, combining this information with other knowledge (using machine learning methods) clearly improves the identification of peptide binding regions within a protein sequence. This approach combining docking with machine learning is primarily a predictor of binding to peptide-binding sites, and is not intended as a predictor of specificity of binding to particular receptors.

## Introduction

Thousands of proteins expressed in cells carry out their specific intracellular and extracellular functions by interacting with each other (protein-protein interactions). These interactions have been acknowledged to play fundamental roles in almost every biological event. Significant biological processes such as protein signalling, trafficking and their synchronised degradation [1], [2], DNA repairing, replication and gene expression [3], [4] require interaction between protein-protein interfaces to perform their tasks. The extent of complexity, co-operativity and diversity for these interactions is enormous, and itself is coordinated by intricate regulatory networks that will ultimately determine the behaviour of biological systems. Many interactions are mediated between the two domains of globular proteins (domain-domain interactions) which tend to be stable (contact surfaces are flat) and require an average size of 1,500–3,000 Å^2^ [5], [6]. Others are intended for fast response to stimuli (domain-motif interactions (DMI)/domain-peptide interactions/peptide-mediated interactions) [7] that occur when a globular domain in one protein recognises a short linear peptide from its corresponding protein partner, creating a comparatively small interface with an average size of 350 Å^2^ [8]. An estimated 15–40% of all interactions in the cell are protein-peptide interactions [4], [9]. These peptide regions are ideal for signalling transduction networks because they are specific, transient and have low-affinity (1–150 μM) [10].

Typically, the peptides involved in DMI or protein-peptide interactions are categorised by a simple sequence pattern, that is, a short linear motif (SLiM) [11], also referred to as linear motifs, minimotifs [12], pre-structured motifs (PreSMos) [13], Eukaryotic Linear Motifs (ELMs) [14] or molecular recognition features (MoRFs) [15]. These peptide regions can vary in their length from 3 to 12 amino acid, typical of SLiMs, through to much longer regions of up to 70 amino acids [16]. In general, SLiMs can be expressed as regular expressions, a consensus motif with specific conserved residues restricted to particular positions recognised by a binding domain, with a set of similar residues or even arbitrary ones at other locations [17]. Structurally, SLiMs are frequently found in disordered regions at protein termini or between domains [18] with the ability to adopt a variety of conformations [15], [19]. SLiMs may also originate from loops within a structured domain, exposing them to potential binding partners including many of the disordered interaction hubs [20], [21] explaining the many functional roles for these regions. The small binding areas which SLiMs constitute result in weak binding [9] making them suitable for short-lived interactions [22]. However, regardless of their short length, these motifs bind their target protein with sufficient strength to establish a functional relationship [23].

Several databases exist to capture instances of SLiMs, for example, Minimotif Miner [12] and the ELM server [14] and a variety of methods are available for novel SLiM discovery. Primarily, these methods which seek to identify over-represented, convergently evolved, motifs in protein sequences [24], [25], however, the motif may be over-represented in a protein by chance [26]. To avoid identifying false positives many methods employ evolutionary information at the local and global level to filter potential SLiM instances [27]. Profile based methods may be able to improve on this but are unable to identify motifs that occur with low frequency or as single occurrences [28]. Recently, a number of *de novo* prediction methods that are not dependent on evolutionary information have been developed [29]–[31]. These methods use the physicochemical properties of the protein sequence and predicted structural features to predict protein binding regions, however, until now there has been no publicly available prediction method which exploits protein-peptide structures available in the Protein Data Bank (PDB) [32].

At present, the estimated number of protein-peptide interactions in the proteome is not reflected in the number of protein-peptide structures available in the PDB. However, the rapid increase in protein structural data in the PDB does provide an excellent opportunity to investigate how this information might be exploited to predict potentially SLiM mediated protein-peptide interactions in a novel manner. As the number of PDB protein structures available is limited, methods that can provide useful information about protein-peptide interactions in the absence of structural information, for example in the case where one of the interactors is unstructured or disordered, are desirable. A possible solution is to use protein-peptide docking in order to infer interaction information, such as the likely binding regions, when a PDB structure is available for only one of the interacting pairs.

Computational docking is a technique that uses protein three-dimensional structural information to predict ligand binding poses. There are two main kinds of docking: protein-ligand docking, where a flexible small-molecule compound is “docked” to a known or suspected protein-binding pocket; and macromolecular docking, where two rigid protein (or other biological macromolecule, such as DNA) structures are docked, with the goal of identifying protein-protein interaction interfaces. Both protein-ligand and macromolecular docking have been used successfully to predict binding poses. Protein-ligand docking is commonly used as an early step in small-molecule drug screening campaigns, where it has been shown to have the ability to accurately retrieve the known binding poses of diverse sets of small molecule-protein complexes, although this does depend somewhat on the protein family, and the characteristics of the binding site [33]. Recently, two high-throughput macromolecular docking experiments have been reported that demonstrated the use of a general docking method to detect interacting partners. Mosca *et al* [34] used docking to identify pairs of proteins that accurately interact with each other in the *Saccharomyces cerevisiae* interactome by sampling from a large set of alternative possibilities. Wass *et al* [35] successfully distinguished between interacting (native) and non-interacting (non-native) protein partners. A disadvantage of macromolecular docking is the requirement for structural data for both proteins. In general, docking programs are not optimised for peptide docking and are most successfully used with small molecules. Unconstrained peptides are flexible and tend to adopt several conformations by rotating within the given search space of the receptor site adding complexity to the docking protocol as the number of rotatable bonds increases. Despite this, peptide docking experiments have recently been used to successfully predict the interaction site of elastin-binding protein and an elastin peptide motif [36], confirming the potential of peptide docking.

Here, we investigated if docking, with AutoDock Vina [37], can be used to identify protein-peptide interactions with the objective of evaluating if the docking score could be used to discriminate a peptide binding region from adjacent non-binding regions within a defined stretch of protein sequence. First, we generated a non-redundant dataset of protein receptor and SLiM containing peptide interacting structures from the ELM database [14]. We then performed high throughput docking of sets of overlapping peptides generated by moving a sliding window along a 50 residue region from the parent protein sequence which is centred around the SLiM containing peptide. We compared these results to those obtained by submitting the same sequences to SLiMPred [30], MoRFpred [29] and ANCHOR [31]. Finally, we trained a bidirectional recurrent neural network (BRNN) to predict the peptide binding region within a protein sequence, using as input the protein sequence, predicted secondary structure, predicted disorder and Vina docking score.

## Results

We evaluated if the Vina docking score could be used to identify peptide binding regions in protein sequences. Specifically, if we have the 3D structure of a peptide binding domain could we predict regions within an unstructured protein sequence that might interact with it?

**Figure 1:**
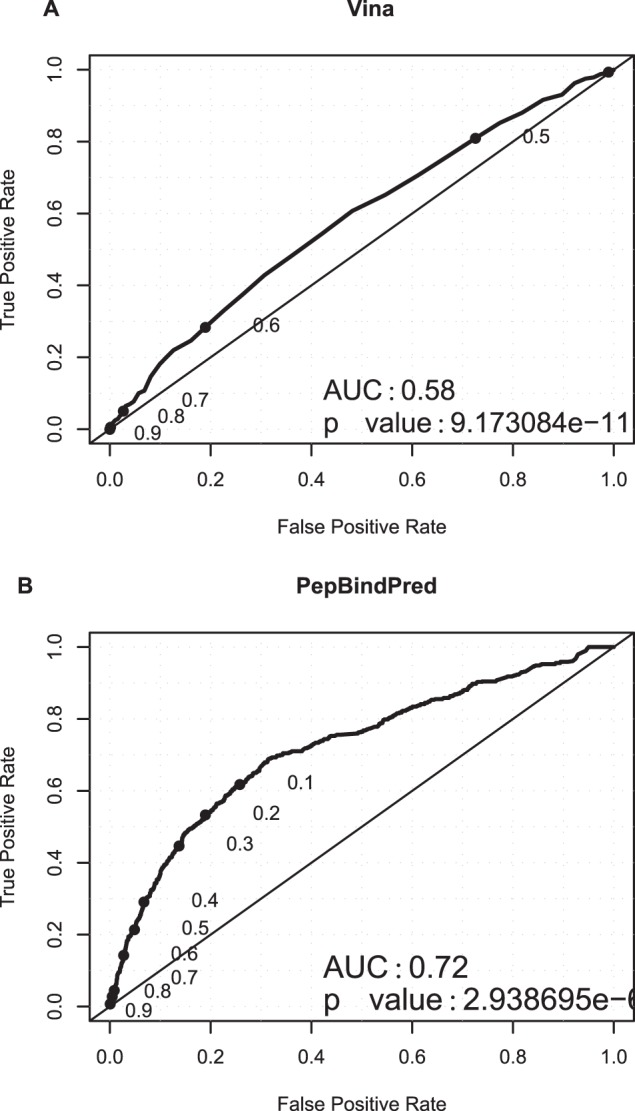
ROC curves – Training set. ROC curves plotting the true positive rate of peptide binding residue identification against the false positive rate, with thresholds for a positive identification decreasing from 1 to 0, tested on the training set of 84 ELM containing examples (A) normalised Vina scores (B) ten-fold cross-validation PepBindPred predictions.

For each of our 84 ELM instances we selected a window of 50 residues around each ELM, where the ELM is in the centre of the region, unless it is found within the 25 residues closest to the N or C terminus, in which case the 50 N or C terminal residues are selected. We prepared sets of overlapping peptides of lengths three residues by sliding a window along these 84 regions of the protein sequence. We docked each tripeptide to the respective PDB receptors. The best Vina score for each peptide was normalised and the results displayed as ROC curves (Figure 1). The results (AUC of 0.58) show only very modest discrimination between peptides involved in binding and non-binding peptides. Given this poor performance we investigated if the docking scores could be used as input to a BRNN and if this would improve the predictive power. We used the same set of 50 residue sequence regions to train seven BRNN in ten-fold cross-validation using a similar approach to that used to train SLiMPred [30]. The predictors were trained using as input the 50 residue sequence, and either predicted secondary structure, predicted disorder or Vina score, or a combination of two, or all three of these features. We plotted ROC curves for the performance of the training set in ten-fold cross-validation for each of the seven predictors and the AUCs are shown in Table 1. The BRNN does in fact improve the predictive power, from an AUC of 0.58 when the Vina scores are assessed, to an AUC of 0.68 using only the Vina score and the sequence to train the BRNN. However, we found that the combination of all three features provided the highest AUC (0.72) (Figure 1), compared to using only secondary structure and disorder predictions without Vina (AUC 0.63). We refer to this method as PepBindPred (Peptide Binding-region Predictor).

**Table 1:** AUC calculated from ROC curves for BRNN trained with seven different input options.

BRNN Input	AUC
Sequence, secondary structure and disorder	0.63
Sequence and secondary structure	0.64
Sequence and disorder	0.64
Sequence and Vina	0.68
Sequence, secondary structure and Vina	0.71
Sequence, disorder and Vina	0.71
Sequence, secondary structure, disorder and Vina	0.72

Predictors trained with either secondary structure, disorder or Vina score along with the protein sequence, or a combination of two, or all three of these features.

**Figure 2:**
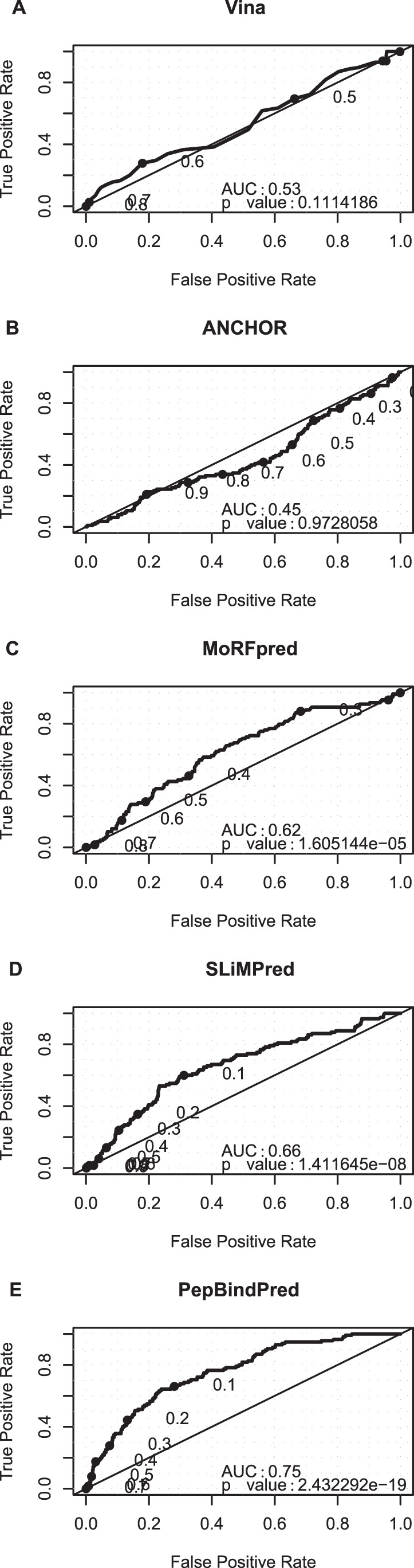
ROC curves – independent test set. ROC curves plotting the true positive rate of peptide binding residue identification against the false positive rate, with thresholds for a positive identification decreasing from 1 to 0, tested on the independent test set of 21 examples (A) Vina (B) ANCHOR (C) MoRFpred (D) SLiMPred (E) PepBindPred.

In order to bench mark PepBindPred we generated an independent test set of 21 disordered sequence regions which are shown to interact with protein receptors in the PDB (see Materials and Methods). We submitted these to PepBindPred, SLiMPred, MoRFpred and ANCHOR, and also compared the results to the Vina docking scores for overlapping tripeptides using the same method as described above. Again we see that the Vina docking scores do not discriminate well between binding and non-binding residues (AUC 0.53) (Figure 2 (A)). While the performances of ANCHOR is not strong (Figure 2 (B)), this is not surprising, since it is designed to identify larger scale regions involved in protein binding, rather than to identify specific residues within such binding regions that bind to peptide-binding domains. MoRFpred aims at shorter sequences (5–25 residues), which may explain why its performance is better (Figure 2 (C)), but less efficient than SLiMPred which targets shorter sequences (Figure 2 (D)). The results for these alternative methods are included as a reminder to use the appropriate computational tool when addressing a specific question, since PepBindPred substantially out performs ANCHOR, MoRFpred, Vina and SLiMPred (AUC 0.75) (Figure 2 (E)).

To test how specific the predictions for a binding region, or SLiM, are to a particular receptor we repeated the predictions for the 21 sequence in the independent test set but we “shuffled” the receptors. That is, we submitted each sequence to the PepBindPred server, however we selected a receptor other than the native receptor, making sure that the new receptor was not in the same class as the native receptor (i.e. care was taken not to submit a SH3 ligand containing sequence to another SH3 domain). The results were almost identical (AUC 0.75). Although the lack of receptor specificity is surprising in some respect, it is also important to remember that it is the nature of many SLiMs to bind with moderate rather than high affinity [38], and that all the receptors here are peptide binding domains, and therefore have some inherent similarity. In the light of this, we conclude that, PepBindPred is a peptide binding region predictor, not a receptor specificity predictor, and users must remember this when interpreting results. The docking information from Vina is providing an additional layer of information that is not available from the primary sequence alone, regarding the propensity of regions to bind to peptide-binding domains. The precise nature of this additional structural information is not clear, and may in future become clearer as the training sets become larger and more informative.

### PepBindPred Web Server

**Figure 3:**
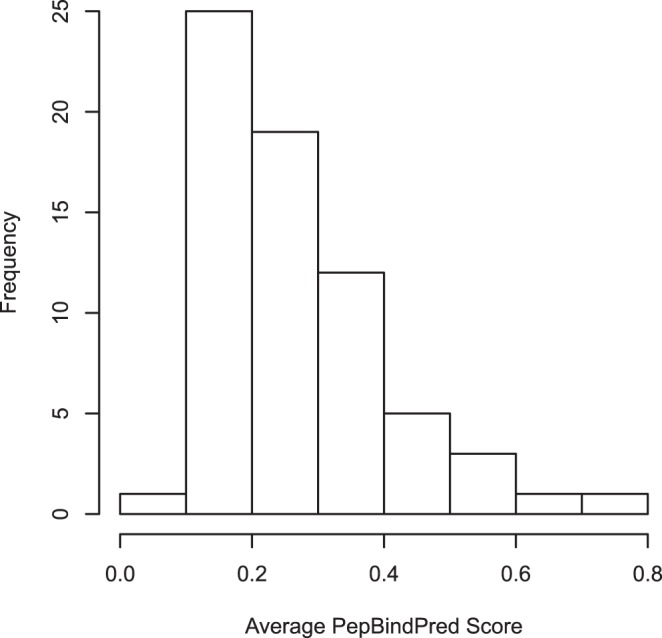
Histogram showing the distribution of PepBindPred scores. The scores have been averaged over the 9 CORNR box motif residues, for each of the 67 instances.

**Figure 4:**
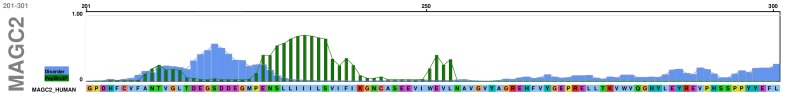
PepBindPred output for Q9UBF1 (MAGC2_HUMAN). Melanoma-associated antigen C2; Motif: LLIIILSVI, residues 229–237.

As an example of the possible use of PepBindPred we used SLiMSearch [39] to identify CORNR box motif instances in the human proteome. 67 instances of the motif (L[ ˆP]2,2[HI]I[ ˆP]2,2[IAV][IL]) were found in 65 Proteins. The ELM server identified two of these as true positives (O75376 and Q9Y618), both of which were ranked highly by SLiMSearch, using conservation analysis. We investigated if PepBindPred would be able to suggest some other potential true positive candidates in this set of CORNR box motif instances. The PepBindPred server takes as input the UniProt [40] identifier of the sequence, the residue to start the search at (we set this to 25 residues before the start of the motif instance), the PDBID and the chain ID (for this example 3N00 and B respectively). We averaged the PepBindPred predictions over the nine motif residues for the 67 instances and have listed them in order in Table 2. The score distribution is skewed, with two of the true positives in the tail (Figure 3), which suggests that some of the other top ranked motif instances could be worth further investigation. The O75376 true positive instance is ranked highest, however this is not surprising as the sequence is in our training set. The second highest ranked sequence, P05160, we think is a false positive as the motif is found in a signal peptide. However the third ranked sequence we believe may be worth further investigation as a possible true positive (Figure 4). This protein, Melanoma-associated antigen C2 (Q9UBF1 (MAGC2_HUMAN)), has a subcellular location of “Cytoplasm. Nucleus.” which is compatible with the function of the CORNR box motif binding to nuclear receptors. The ROC curve for the independent test set results (Figure 2 (E)) suggests that 0.4 is a good threshold to choose as a cut-off as the false positive rate is still low (approximately 3%), while allowing for the capture of more true positives (approximately 20%, compared to <10% at a threshold of 0.5).

**Table 2:** PepBindPred predictions, averaged over the nine motif residues, and evolutionary conservation p-value for the motif instance.

UniProt AC	Motif	PepBindPred Score	Evolutionary conservation p-value
O75376*	LADHICQII	0.715	0.048
P05160	LTFIIILII	0.680	0.969
Q9Y618*	LEAIIRKAL	0.557	0.026
Q9UBF1	LLIIILSVI	0.543	1
Q96BZ9	LIDIILLIL	0.520	0.208
Q8NI22	LINIIDGVL	0.486	0.214
Q07325	LLGIILLVL	0.467	0.396
Q5SVZ6	LKLIIENIL	0.434	0.329
Q96AH8	LKLIIVGAI	0.425	0.75
Q9HAU8	LTFIISSIL	0.401	0.436
Q9NRU3	LEDIIEEII	0.384	0.393
P53618	LMTIIRFVL	0.363	0.321
O75376*	LEDIIRKAL	0.362	0.044
Q8IWF6	LRTHIDAII	0.350	0.197
Q8NHV5	LFFIIMGII	0.341	1
Q9UPM8	LRLHIIEII	0.338	0.3
Q9Y618*	LAQHISEVI	0.335	0.055
Q96N64	LDHIIEDAL	0.333	0.562
O95477	LSRIIWKAL	0.332	0.614
O00273	LASHILTAL	0.327	0.546
Q7Z3J2	LQLIIKKVI	0.325	0.096
Q09161	LNYHIVEVI	0.306	0.799
Q08AE8	LGIIIYKAL	0.294	0.164
Q8TDJ6	LNNHIHDIL	0.287	0.115
P07384	LYQIILKAL	0.283	0.518
Q5MIZ7	LYEIIRGIL	0.282	0.295
Q8TDR0	LHDIITEVI	0.282	0.43
Q96PN6	LKNIITVVI	0.276	0.645
Q8TCG5	LGQHIEDAL	0.274	0.294
Q6ZMV5	LYEIIKGIL	0.272	0.321
Q8IX04	LQYIITNVL	0.267	0.539
Q93100	LVIHIGWII	0.267	0.81
Q8TDL5	LKNIITEII	0.267	0.617
Q9C093	LVDIIVNAI	0.266	0.086
Q7RTX7	LARIIRVIL	0.262	0.345
Q9UIA9	LVYIIGAVI	0.257	0.104
Q8NEG5	LCKHICWVL	0.257	0.114
Q9Y6X3	LLGHIFYVL	0.232	0.885
Q8IZQ1	LAQIILDAI	0.221	0.708
P35556	LNNHIRYVI	0.216	0.145
Q14185	LLSHILEVL	0.209	0.067
O95801	LKAIIRGAL	0.199	0.732
A6NHC0	LYQIIRKAL	0.193	0.817
P56192	LGNIIGCVL	0.189	0.542
A6NES4	LTSIIVAVI	0.184	1
O95714	LCTHIGDIL	0.183	0.034
Q8NF50	LVGIILDAL	0.182	0.629
O95450	LGAHINVVL	0.174	0.364
Q8N485	LRHIIAQVL	0.174	0.898
Q8TCG1	LKMHIAKIL	0.173	0.55
Q6R327	LDHIIQKAI	0.162	0.067
Q5T215	LCGIIRGAL	0.160	0.131
Q8WZ26	LSTHICVVL	0.159	1
P51124	LTFHIKAAI	0.158	0.658
Q99698	LNSIIDQAL	0.156	0.672
Q562E7	LSDITYYVY	0.156	0.94
Q9UJ70	LGRHIVAVL	0.153	0.136
Q0VDD8	LDKHIKSAI	0.152	1
Q8N1T3	LFGIIASVL	0.151	0.326
P17655	LFKIIQKAL	0.148	0.804
P52743	LHVIIDFIL	0.147	1
Q6PGP7	LEDIIGFAL	0.146	0.128
Q13572	LLNHIATVL	0.134	0.551
O15072	LGVHINVVL	0.128	0.362
Q8WXS8	LGVHINIAL	0.110	0.585
Q9UG01	LVEHITAAL	0.107	0.082
P30307	LGGHIQGAL	0.061	0.072

PepBindPred predictions, averaged over the nine motif residues, and evolutionary conservation p-value for the motif instance, calculated using SLiMSearch [39], for the 67 CORNR box motif instances in the human proteome. Scores closer to 1 indicate that PepBindPred is more confident that regions is peptide binding, whereas SLiMSearch p-values closer to 0 indicate that the motif is more likely to be a true positive due to conservation. Two of the sequences have two instances of the motif, O75376 and Q9Y618. *True positives identified on the ELM server.

There are many other biological application of this method beyond the example given. PepBindPred is computationally more intensive than ANCHOR, MoRFpred or SLiMPred, so we would suggest using one, or all, of these methods on your protein, or proteins, of interest first to predict peptide binding regions. PepBindPred can then be used to refine the regions identified by these methods, as it has been shown to be more accurate than any of these methods individually. PepBindPred is especially useful where some experimental information is available which would lead the user to believe that the sequence of interest binds to, or interacts with, a particular structured protein domain for which there is a PDB structure available.

## Discussion

Disordered regions of proteins often bind to structured domains, mediating interactions within and between proteins. We have presented a computational analysis of the performance of peptide docking with AutoDock Vina to assess if the Vina docking scores could be used to predict protein-peptide interactions. As Vina is designed for small-molecule docking with a restriction on the number of rotatable bonds (≤32), it is generally assumed that it is not suitable for docking peptides which have many more internal degrees of freedom. Previously, however, we have shown that there is a correlation between the Vina docking scores of dipeptides with ACE and experimentally determined ACE inhibition (IC50) [41]. Extensive preliminary work [42] which attempted to dock the full peptide sequence to the peptide binding domain showed that there is a direct correlation between the peptide length and the number of rotatable bonds in the peptide. As the number of rotatable bonds increase so does the RMSD between the docked peptide pose and the native peptide pose. Following this analysis we attempted to dock much shorter peptide fragments (2–5 residues in length) to the peptide binding domain and determined that tripeptides gave the best results [42].

Here, we investigated the binding of known SLiM-containing peptides from within disordered protein regions to peptide binding domains and investigated the docking of adjacent overlapping peptides from the peptide’s protein sequence. We found that we were unable to discriminate between binding and non-binding peptide regions based on the Vina docking scores alone in this context. We then trained a BRNN using the peptide sequence, predicted secondary structure, predicted disorder and Vina score as inputs. Our analysis shows that when the Vina docking score was used as an input to our BRNN, in combination with predicted secondary structure and disorder, the AUC increased from 0.63 to 0.72, compared to using only predicted secondary structure and disorder. We have shown that this method, PepBindPred, performs better on an independent test set than three other publicly available *ab initio* methods, ANCHOR, MoRFpred and SLiMPred. This approach emphasises the potential for including structural information when developing methods for refining the location of peptide binding residues within disordered protein regions.

In this study we have evaluated only one docking method, AutoDock Vina. The Vina docking program has many advantages, it is easy to install and run locally, it is extremely fast and is very suitable for high-throughput docking which is essential for implementation as a web server of this kind. It is possible, however, that other methods, for example, DynaDock [43], which has been developed more specifically for the docking of peptides into flexible binding sites, may provide better results in individual cases. Unfortunately, this method is slower than Vina, so it is not suitable for high-throughput docking in this context. Another method, the Rosetta FlexPepDock server [44], refines docking conformations against a given PDB file and an estimated peptide conformation, however, FlexPepDock is computationally intensive as the protocol samples a significant conformational space and therefore, similar to DynaDock, is also not suitable for our use.

We are not entirely sure why the Vina docking score is unable to discriminate between binding and non-binding residues. The Vina scoring function (see [37] for details) uses a weighted sum of interactions to predict a binding pose. Values for the weights were determined by fitting the scoring function to the PDBBind refined dataset. It is possible that the dataset used to fit the scoring function is biased towards small molecules rather than peptides. This could possibly explain why the binding site cannot be predicted using only the Vina docking score. Furthermore, although there is no fixed limit, it has been shown that Vina can handle ligands with up to 32 rotatable bonds [37], and tripeptides would be on the high end of this scale. Finally, even with small peptides, in this case tripeptides, the search space is large, and difficult to search, although we never exceed the maximum search space recommended (i.e 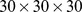
Å).

Docking of tripeptides was chosen by us as a compromise between computational efficiency and potential informativeness as tripeptides appeared to perform slightly better than other longer peptide fragments in preliminary experiments [42]. Using longer peptides would likely require alternative strategies starting with many more initial configurations of the peptides, in order to adequately sample the conformational space prior to seeking to identify minima. This would substantially increase the computational burden of docking peptides along the sequence. It is also possible that alternative docking methods or scoring functions may contribute to improved performance, unfortunately this is beyond the scope of this work at present, however, they will be of interest for future work. While it is possible that the initial docking may be improved over that used, we consider it likely that the kind of machine learning step we introduced here is still likely to improve on the docking in discriminating between binding and non-binding adjacent regions along a protein sequence.

PepBindPred is available as part of our web server for SLiM discovery and annotation. The user submits the protein sequence that they wish to predict peptide binding regions within, and the PDB ID of the protein structure they wish to predict interactions with. At present the PDB structure is required to have a bound peptide, and the user must provide the chain ID of the bound peptide so that the search space for Vina can be defined around it. Predictions take approximately one hour per protein. Our server is freely available for academic users at http://bioware.ucd.ie/pepbindpred.

## Methods

### Datasets

1,358 true positive LIG (ligand binding) ELM instances were downloaded from the ELM server [14]. 208 of these were annotated as having a structure in the PDB. Using the FASTA sequence of these 208 ELM containing sequences we predicted the disorder using IUPred [45]. We retained 135 sequence which had an average IUPred score of >0.5 (i.e. disordered) over a 50 residue window around the ELM. We internally reduced this set to make it non-redundant using BLAST [46] with an e-value of 0.001, to less than 30% sequence similarity between any two sequences, leaving 122 sequences. In some cases the exact ELM motif was not found in the bound peptides of the PDB structures and these structures were removed. The PDB chain ID of the 84 matches that were found were noted and used to define the centre of search space for Vina, which was then extended to cover the bound peptide [37]. The peptide chain was then removed from the PDB structure in order to allow for docking to this site. AutoDock 4.2 [47] was then used to prepare the PDB files as “receptors” for Vina.

All possible peptides were generated by scanning a sliding windows of three residues along the 50 residue section of the 84 ELM containing protein sequence. Peptides were converted into the SMILES format using CycloPs [48], and from SMILES to PDB format using Open Babel [49]. AutoDock 4.2 [47] was used to prepare the peptides as ligand files for Vina.

An independent test set was generated in a similar manner using the sequences in the SLiMPred “SteinAloy” independent test set, which was derived from analysis of peptide-mediated interactions within PDB structure by Stein and Aloy [50], see [30] for more details. The original dataset was redundancy reduced to less that 30% sequence similarity to our training set leaving 46 sequences with a peptide region which is known to interact with a PDB structure, 21 of these are in regions which are predicted to be disordered.

Anchor [31], MoRFpred [26] and SLiMPred [30] were used to predict protein binding regions, and Distill [51] was used to predict secondary structure, for the full protein sequence. The predictions in the 50 residue window around the ELM were then extracted.

### Analysis of Docking Results

The output generated by Vina for each peptide was processed and the binding conformation having the lowest binding affinity (i.e the top ranked Vina docking score) was selected for further investigation. To evaluate the ability of Vina to discriminate between binding peptides and adjacent non-binding peptides extracted from the same protein sequence, we measure the TPR and FPR. First, we normalise the Vina docking scores for each peptide so they fall between 0 and 1: 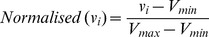
(1)where 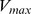 is the absolute value of the minimum Vina binding score (i.e. 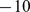) and 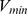 is zero. We measure the sensitivity or true positive rate (TPR) and specificity or false positive rate (FPR) as we increase the discrimination threshold from 0 to 1. We plot the TPR against the FPR as Receiver Operating Characteristic (ROC) curves, which are calculated as follows:



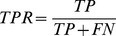


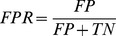
(2)


R [52] was used to plot the curves, and calculate the AUC. The AUC is a number between 0 and 1 inclusive, where 0.5 indicates a random model and 1 is perfect, which is equivalent to the probability that a randomly chosen positive instance will rank higher than a randomly chosen negative instance [53].

### Implementation of BRNN

BRNN have recently been used successfully for the prediction of peptide binding regions using the protein sequence, predicted secondary structure, structural motifs, solvent accessibility and disorder as inputs [30]. We use a similar model here using the protein sequence, predicted secondary structure, predicted disorder and Vina score as input. See [54] for a detailed explanation of the BRNN model.

These networks take the form: 
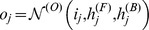


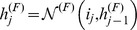


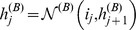


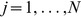
(3)where 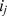 (respectively 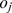) is the input (respectively output) of the network in position 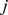, and 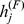 and 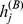 are forward and backward chains of hidden vectors with 
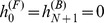
. We parametrise the output update, forward update and backward update functions (respectively 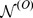, 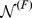 and 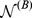) using three two-layered feed-forward neural networks.

Input 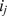 associated with the 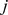-th residue contains primary sequence information and predicted structural information (secondary structure, disorder and Vina score):
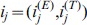
(4)where, assuming that 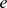 units are devoted to sequence, and 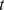 to structural information:
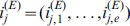
(5)and:



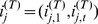
(6)Hence 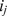 contains a total of 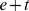 components.

We use 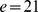: the 20 standard amino acids are considered, while the 21st input encodes the length of the sequence. We use 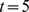 for representing structural information. The first three structural input units contain the predicted three-class secondary structure representing the predicted probability of the 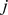-th residue belonging to either helix, strand or coil and the final two inputs are the predicted disorder and Vina score. Hence the total number of inputs for a given residue is 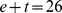.

### Training, Ensembling

Training is conducted by ten-fold cross-validation, i.e. ten different sets of training runs are performed in which a different tenth of the overall set is reserved for testing. The ten fifths are roughly equally sized, disjoint, and their union covers the whole set. The training set is used to learn the free parameters of the network by gradient descent. Three models are trained independently for each fold and ensemble averaged to build the final predictor. Differences among models are introduced by varying the size of the input window considered by network from 7 to 9 to 11 residues. 10,000 epochs of training are performed for each model and the learning rate is halved every time we do not observe a reduction of the error for more than 50 epochs.
